# Structural analysis of variant of *Helicobacter pylori* MotB in its activated form, engineered as chimera of MotB and leucine zipper

**DOI:** 10.1038/s41598-017-13421-0

**Published:** 2017-10-18

**Authors:** Daniel A. Andrews, Yuri E. Nesmelov, Matthew C. Wilce, Anna Roujeinikova

**Affiliations:** 1 Department of Microbiology, Monash University, Clayton, Victoria, 3800 Australia; 2 Department of Physics and Optical Science, University of North Carolina at Charlotte, Charlotte, NC USA; 3 Department of Biochemistry and Molecular Biology, Monash University, Clayton, Victoria, 3800 Australia; 4 Infection and Immunity Program, Monash Biomedicine Discovery Institute, Monash University, Clayton, Victoria, 3800 Australia

## Abstract

Rotation of the bacterial flagellum is powered by a proton influx through the peptidoglycan (PG)-tethered stator ring MotA/B. MotA and MotB form an inner-membrane complex that does not conduct protons and does not bind to PG until it is inserted into the flagellar motor. The opening of the proton channel involves association of the plug helices in the periplasmic region of the MotB dimer into a parallel coiled coil. Here, we have characterised the structure of a soluble variant of full-length *Helicobacter pylori* MotB in which the plug helix was engineered to be locked in a parallel coiled coil state, mimicking the open state of the stator. Fluorescence resonance energy transfer measurements, combined with PG-binding assays and fitting of the crystal structures of MotB fragments to the small angle X-ray scattering (SAXS) data revealed that the protein’s C-terminal domain has a PG-binding-competent conformation. Molecular modelling against the SAXS data suggested that the linker in *H*. *pylori* MotB forms a subdomain between the plug and the C-terminal domain, that ‘clamps’ the coiled coil of the plug, thus stabilising the activated form of the protein. Based on these results, we present a pseudo-atomic model structure of full-length MotB in its activated form.

## Introduction

Motility by flagellar motor is essential for the survival and virulence of many pathogenic bacteria associated with human and animal diseases^1,2^. Unlike other biological macromolecular systems that generate mechanical motion (e.g. myosin in muscles), the bacterial flagellar motor (BFM) does not directly use the energy of ATP hydrolysis. Instead, rotation is powered by the energy of the electrochemical gradient across the cytoplasmic bacterial membrane^3^. The BFM functions as a membrane-embedded rotary engine. The core component of its rotor, the rod, is surrounded by several rings^4–6^. In most flagellated bacteria, the force that rotates the rod is generated by the proton influx through several circumferentially positioned peptidoglycan (PG)-anchored MotA_4_MotB_2_ stator complexes^7^. Each MotA_4_MotB_2_ complex is believed to contain two proton-conduction channels that span the cytoplasmic membrane^8^.

Little is known about the mechanism by which the force-generating component, the stator, assembles and functions. It is now clear that, like machines invented by humans in the macroscopic world, the BFM contains highly coordinated moving parts that become replaced as they ‘wear out’ (get damaged) or become misaligned. For instance, the stator proteins that were previously thought to form a stable ring, dissociate every ~0.5 min, swapping with new ones from a pool of ‘spare parts’ (free stator units diffusing in the membrane)^9^. Furthermore, the number of the stator complexes bound to the motor was shown to change according to the torque generation requirements of the bacterium^10,11^. However, our understanding of the mechanism of the stator assembly and activation is limited to an inferred localisation of the stator in low-resolution tomographic reconstructions of the entire motors^12,13^, low-resolution single-particle reconstruction of the single stator unit in its inactive form^14^, protein proximity as deduced from crosslinking and molecular genetics studies^4,15–19^, overall topology inferred from the crosslinking and gel-filtration studies^7,8,20^, and the crystal structures of the periplasmic part of MotB^21–23^.

**Figure 1:**
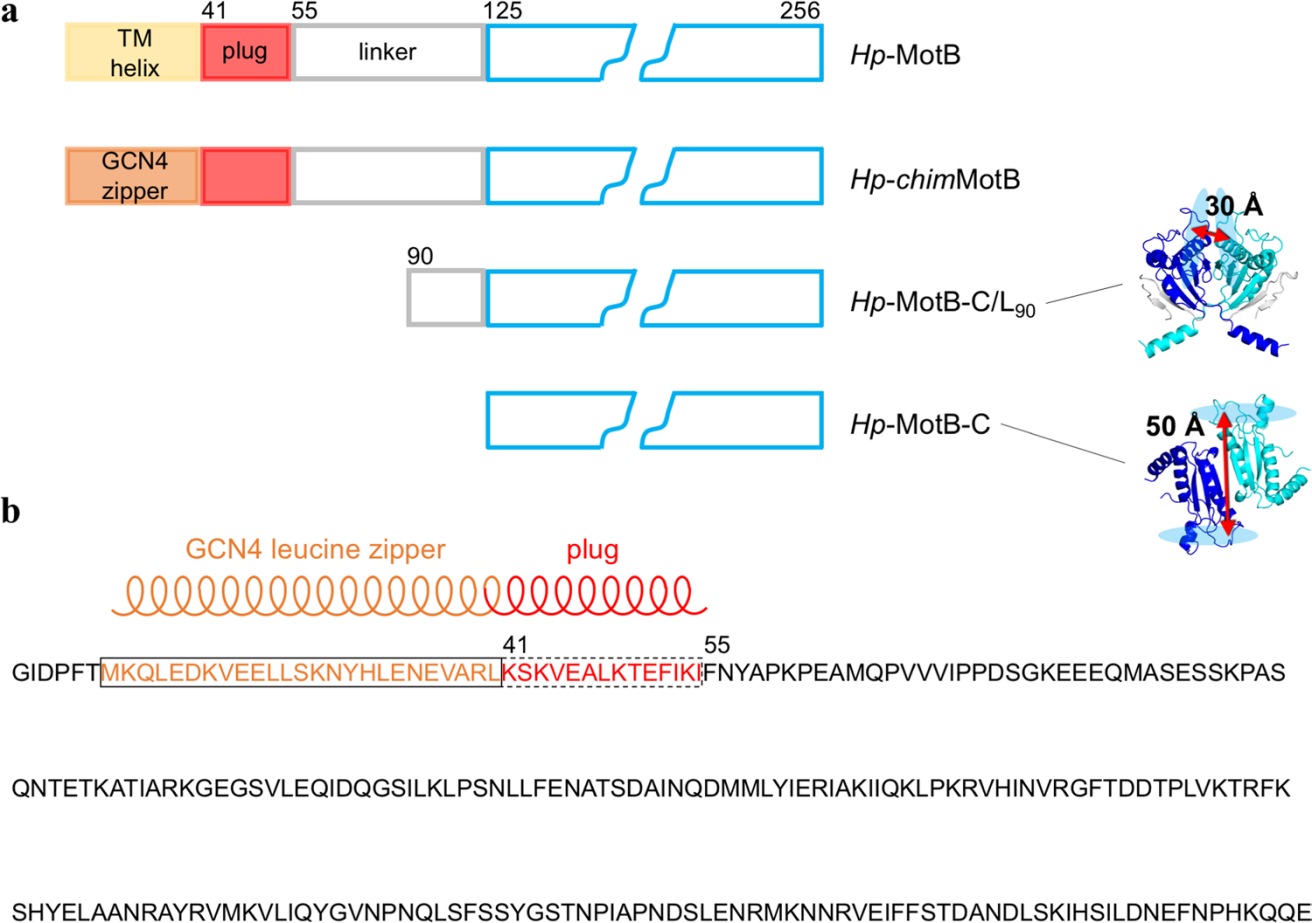
MotB constructs used in this study. (**a**) Schematics of native *H*. *pylori* MotB and its soluble variants. The peptidoglycan-binding domain is drawn in blue. The crystal structures of the dimers of *Hp*-MotB-C/L_90_^23^ (PDB ID: 3S0H) and *Hp*-MotB-C^21^ (PDB ID: 3CYP) are shown, illustrating the different relative orientation of the two monomers and different spacing between the glycan-binding grooves (shown as light-blue ovals). The residues of the two peptidoglycan-binding domains are coloured blue and cyan; the linker residues in the crystal structure of *Hp*-MotB-C/L_90_ are coloured grey. (**b**) The design of the chimeric variant *Hp*-*chim*MotB^31^ that mimics full-length MotB in its activated (plug as a coiled coil) form. The transmembrane (TM) helix in this chimera is replaced with the GCN4-derived leucine zipper motif; N-terminal GIDPFT is the cloning tag.

MotB is anchored to the cytoplasmic membrane and to MotA *via* its N-terminal hydrophobic transmembrane (TM) α-helix. The C-terminal domain of MotB (MotB-C), connected to the N-terminal helix *via* a linker, tethers the stator complex to PG of the cell wall around the rotor, thereby immobilising the stator ring^23–25^. Analysis of the crystal structure of the complex between MotB-C from *Helicobacter pylori* (*Hp*-MotB-C, residues 125–256) and a small PG fragment^21^ suggested that two sugar chains of PG can bind simultaneously to the two symmetry-related sugar binding grooves of the MotB dimer separated by 50 Å (Fig. 1a), whereas the concave surface at the dimer interface is a likely binding site of a peptide cross-bridge of PG. In line with this analysis, it has been subsequently shown that MotB dimerisation *via* MotB-C is essential for its function^22,26^.

MotA_4_MotB_2_ complexes are assembled in the membrane and do not conduct ions until they incorporate into the motor^27,28^, whereupon the linker region of MotB is thought to separate from the core domain and adopt a more extended conformation^22,23^. These inactive MotA/B complexes diffusing in the membrane do not bind strongly to the cell wall^9^, in agreement with the observation that MotB (in the membrane pool) could not be co-isolated with PG^26^. To explain this phenomenon, we have recently shown that the PG anchor of the *H*. *pylori* stator can adopt two distinctly different dimeric conformations, only one of which (represented by the structure of *Hp*-MotB-C) is spatially primed for binding to the cell wall^23^ (Fig. 1a). In the alternative conformation, represented by the structure of the truncated variant comprising the PG-binding domain and part of the linker (*Hp*-MotB-C/L_90_. residues 90–256), the relative orientation of the two monomers is different from *Hp*-MotB-C, the linker is folded against the conserved core domain, and the two glycan-binding grooves are separated by no more than 30 Å^23^ (Fig. 1a). Since the average distance between the adjacent glycan chains in PG is 50 Å^29,30^, the alternative conformation observed in *Hp*-MotB-C/L_90_ is thought to represent inactive (not optimal for binding to PG) form of the anchor^23^.

Apart from enhanced interaction with the PG, activation of the stator also involves the opening of the proton channel through association of the two plug helices in the periplasmic region of the MotB dimer into a parallel coiled coil^28^. The detailed structural and mechanistic studies of the conformational switch that activates new stator units to couple proton flow with motor rotation, have been significantly hindered by lack of a procedure to produce full-length membranous MotB in a stable and active form. Consequently, all previous structural studies of MotB used its truncated variants^21–23^. Significant cross-species diversity of the resulting structures and the mutagenesis-derived conclusion that most of them, if not all, represent an inactive conformation, have been some of the inherent complications of such an approach.

We have recently overcome this limitation by developing a novel approach that allows production of milligram amounts of a close structural mimic of full-length MotB from *H*. *pylori* - a soluble chimeric variant (*Hp*-*chim*MotB) in which the helical TM domain was replaced with a water-soluble leucine zipper (dimerisation) motif derived from the yeast transcription factor GCN4^31^ (Fig. 1). The TM helices of the two native MotB molecules in the stator complex associate into a parallel symmetric dimer that resembles an α-helical coiled coil, both in the presence and absence of MotA^8^. The C-terminal extension of the MotB TM helix, the plug, also forms a parallel coiled coil when the proton channel is open^28^. Replacing the TM helix with a soluble α-helical coiled coil (GCN4 zipper) in register with the coiled coil heptad motif in the plug produced a soluble protein *Hp*-*chim*MotB in which the two plug helices form a coiled coil extension of the leucine zipper. This design locks the plug in a parallel coiled coil state, mimicking the open (active) state of the stator. The biochemical and biophysical analysis of the resultant protein showed that it was properly folded, stable, behaved as a monodisperse dimer at low pH, and had molecular dimensions close to those expected for native MotB^31^, suggesting that *Hp*-*chim*MotB is a suitable model system for structural studies aimed at the detailed characterisation of the activated form of MotB. Here, we report characterisation of the in-solution structure and properties of *Hp*-*chim*MotB using a combination of fluorescence resonance energy transfer measurements (FRET), PG-binding assays and fitting of the crystal structures of MotB fragments to the small angle X-ray scattering (SAXS) data, and present a pseudo-atomic model structure of full-length MotB in its activated form.

## Results

### *Hp*-MotB-C and *Hp*-*chim*MotB, but not *Hp*-MotB-C/L_90_, bind PG

**Figure 2:**
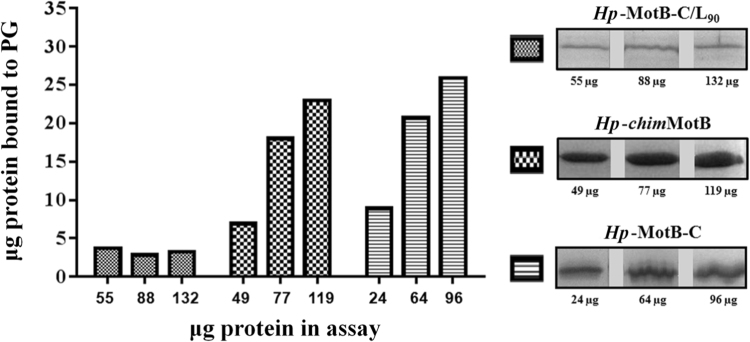
Binding of *H*. *pylori* MotB variants to *H*. *pylori* PG *in vitro*. Purified proteins were incubated with PG isolated from *H*. *pylori* 26695 as described in Materials and Methods. Control incubations were carried out without PG (data not shown). Proteins pulled down together with insoluble PG were analysed and quantified by SDS-PAGE. The amount of protein bound to PG, shown in the bar graph (left panel), was calculated by subtracting the amount of protein recovered from the insoluble fraction in the absence of PG, from the amount of insoluble protein retrieved in the presence of PG. The experiment was carried out twice, and the results averaged. (right panel) Bands on the SDS-PAGE gel showing the protein pulled down together with insoluble PG for three different concentrations of *Hp*-*chim*MotB, *Hp*-MotB-C and *Hp*-MotB-C/L_90_. Each of the three proteins shown was analysed on a separate SDS gel. The three gels were stained/destained together in the same solution, and the bands were cropped and grouped to produce the λure.

To better understand the structural basis of the interaction between *H*. *pylori* MotB and PG, potential association of MotB variants *Hp*-MotB-C, *Hp*-*chim*MotB and *Hp*-MotB-C/L_90_ (Fig. 1a) with PG *in vitro* was assessed using a semi-quantitative pull-down assay with PG sacculi isolated from wild-type *H*. *pylori* 26695. Although partial, aggregation-induced precipitation was observed for all three variants in the absence of PG (~15% for *Hp*-MotB-C, ~35% for *Hp*-*chim*MotB and ~5% of *Hp*-MotB-C/L_90_), the amount of *Hp*-MotB-C and *Hp*-*chim*MotB recovered in the pellet fraction was far higher in the presence of PG, indicating that both proteins bound to isolated PG. In contrast, *Hp*-MotB-C/L_90_ displayed only background-level binding to PG under similar experimental conditions (Fig. 2), as the amounts of protein recovered from the insoluble fraction in the presence and absence of PG were small (3–7%) and comparable. This result is in line with the previous structural studies on *Hp*-MotB-C and *Hp*-MotB-C/L_90_^21,23^, which suggested that *Hp*-MotB-C adopts an active (competent for binding PG) conformation, whilst the linker region of *Hp*-MotB-C/L_90_ stabilises the latter in a distinctly different form that is not competent for binding to PG.

To assess the relative affinities of *Hp*-MotB-C and *Hp*-*chim*MotB to *H*. *pylori* PG, the pull-down assays were performed using a concentration series of each protein with a fixed amount of PG. Although due to multiple wash steps and partial background protein aggregation this technique does not allow reliable estimation of the dissociation constants, it does provide a means to compare affinities of different proteins. As Fig. 2 illustrates, the amounts of *Hp*-*chim*MotB and *Hp*-MotB-C pulled down with PG showed similar increases as the amount of the protein in the assay was increased. This observation indicates that *Hp*-*chim*MotB’s affinity to PG is close to that of *Hp*-MotB-C. Therefore, it can be concluded that the C-terminal PG-binding domain of *Hp*-*chim*MotB likely adopts a conformation similar to that of *Hp*-MotB-C rather than *Hp*-MotB-C/L_90_.

### Time-resolved fluorescence resonance energy transfer (TR-FRET) measurements indicate that the C-terminal domain of *Hp*-*chim*MotB adopts the *Hp*-MotB-C conformation

To characterise *Hp*-*chim*MotB structural states in solution and relate them to the conformations observed for the C-terminal domain of MotB in the crystal^21,23^, ‘the molecular ruler’ application of FRET was used. To facilitate site-directed attachment of fluorescent probes to *Hp*-*chim*MotB, a unique cysteine (Cys146) was introduced at a non-conserved position on the protein surface (there are no other cysteines in *Hp*-*chim*MotB). The position was chosen in such a way that the calculated distance between the two cysteines in the dimer would differ significantly between the two alternative conformations seen in the crystal (>60 Å in the *Hp*-MotB-C/L_90_ structure and approximately 44 Å in the *Hp*-MotB-C structure). The measured interprobe distance was 46.6 ± 6.2 Å (Supplementary Fig. S1), consistent with the conformation observed in the crystals of *Hp*-MotB-C and in agreement with the results of the PG-binding assay which demonstrated that the chimera and *Hp*-MotB-C have similar affinities for PG.

### Structural parameters derived from *Hp*-*chim*MotB SAXS data

#### Hp-chimMotB is an elongated dimer in solution

**Figure 3:**
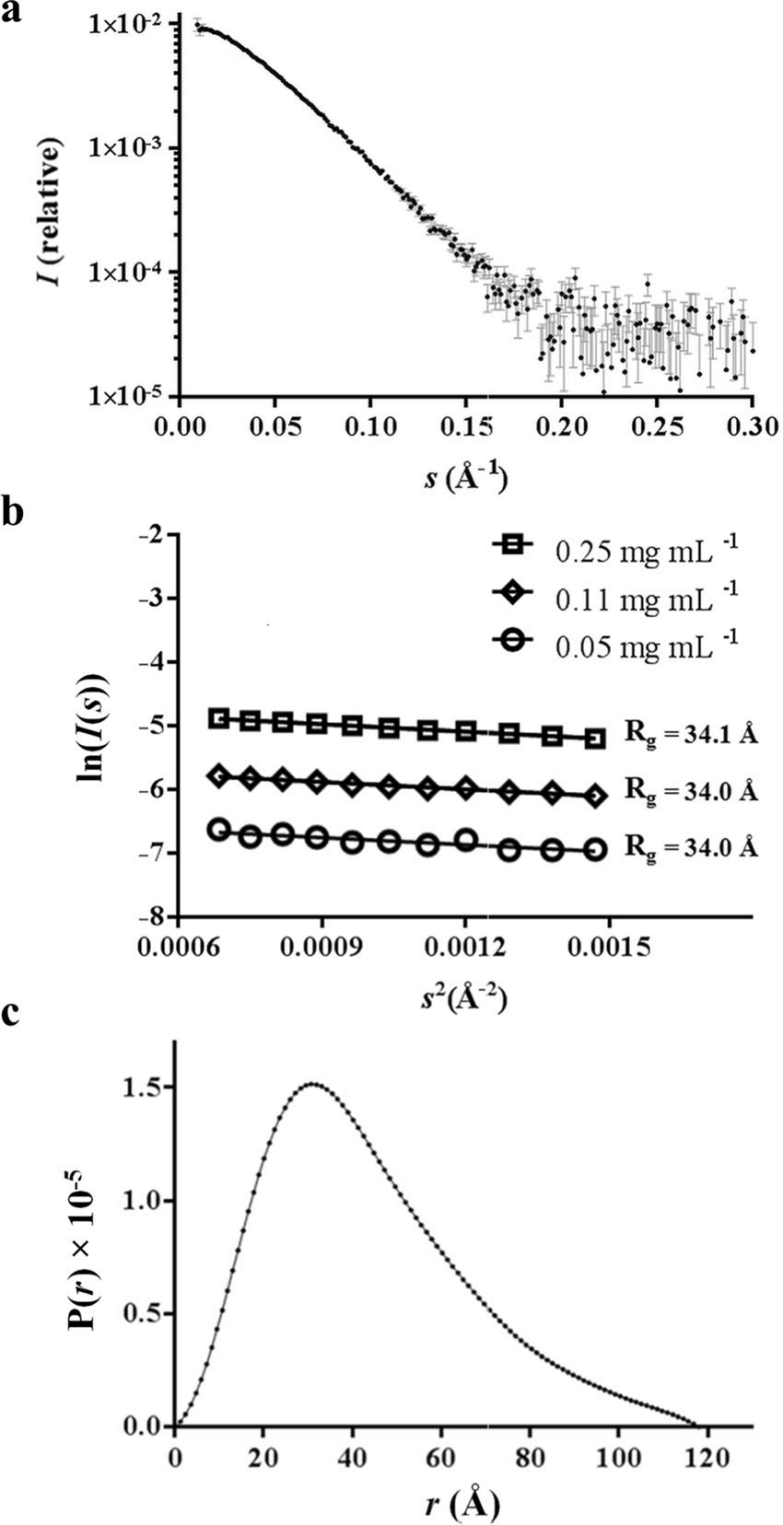
Analysis of *Hp*-*chim*MotB small angle X-ray scattering (SAXS) data. (**a**) 0.25 mg mL^−1^ *Hp*-*chim*MotB scattering intensities plotted on the logarithmic scale as a function of scattering vector magnitude (0.01–0.30 *s*). Data points and error bars are represented as black dots and grey lines, respectively. (**b**) The Guinier region of the 0.25, 0.11, and 0.05 mg mL^−1^ *Hp*-*chim*MotB SAXS data, where *sR*_*g*_ is less than 1.3. (**c**) P(*r*) function calculated from the 0.25 mg mL^−1^ *Hp*-*chim*MotB SAXS data (0.026–0.238 *s*) using a *D*_*max*_ of 118 Å.

SAXS experiments provide time- and ensemble-dependent average geometric parameters of the scattering particles in solution. An indirect Fourier transform of the scattering intensities (*I*(*s*)) produces a P(*r*) function which represents the distance between the electron pairs within the protein as interatomic vectors. GNOM^32^ was used to generate a P(*r*) function from the *Hp*-*chim*MotB 0.25 mg mL^−1^ SAXS data (0.026–0.238 *s*, where *s* is the magnitude of the scattering vector) by varying the P(*r*)_max_ and restraining P(*r*)_min_ to 0 (the experimental SAXS curve and the calculated P(*r*) function are shown in Fig. 3). The maximum dimension of the molecule (*D*_*max*_) was taken as the value that yielded a plausible solution with the largest ‘total score’, while allowing the P(*r*) function to gradually approach 0 at P(*r*)_max_. The radius of gyration (*R*_*g*_) and forward scattering (*I*(0)) values were derived from the second moment of the P(*r*) function and the area underneath the curve, respectively^33^ (Table 1). The P(*r*) function yielded the values of 34.6 ± 0.2 Å for *R*_*g*_ and ~118 Å for *D*_*max*_, and exhibited an asymmetric curve typical for molecules that are moderately elongated in solution (Fig. 3c). *R*_*g*_ and *I*(0) values were also calculated from the Guinier region of the *Hp*-*chim*MotB SAXS data, which remained linear up to *sR*_*g*_ = 1.3 (Fig. 3b). The P(*r*) functions and Guinier analysis of the 0.25, 0.11, and 0.05 mg mL^−1^ *Hp*-*chim*MotB SAXS data gave similar *R*_*g*_ and *I*(0) values (Table 1, Fig. 3b). In addition, for each concentration, estimations of *Hp*-*chim*MotB’s MW from both the *I*(0) (Guinier- and P(*r*)-derived) and the Porod volume^34^ were close to the theoretical molecular mass of the *Hp*-*chim*MotB dimer calculated from the amino acid sequence (Table 1). Taken together, these results indicate that *Hp*-*chim*MotB forms a moderately elongated dimer free of aggregation and interparticle interference across the analysed concentrations. The maximum dimension of the dimer estimated by using this approach (~118 Å) was close to the expected size of MotB in its active conformation (*i*.*e*. long enough to reach the PG layer)^22^.

**Table 1:** Parameters derived from the SAXS analysis of *Hp*-*chim*MotB.

Protein concentration, mg mL^−1^	0.05	0.11	0.25
*I*(0) × 10^−3^ from Guinier plot, cm^−1^	1.6	3.9	9.7
*I*(0) × 10^−3^ from P(*r*), cm^−1^	1.6	3.9	9.6
*R*_g_ from Guinier plot, Å	34.0	34.0	34.1
*R*_g_ from P(*r*), Å	32.4	34.0	34.6
*Dmax*, Å	118	118	118
Porod volume × 10^3^ (Å^3^)	110.3	109.1	106.5
Theoretical MW (dimer), kDa	56.1	56.1	56.1
MW from Guinier plot, kDa	56.2	57.7	58.4
MW from P(*r*), kDa	54.2	56.9	57.9
MW from Porod volume, kDa	64.9	64.1	62.6

#### SAXS data on Hp-chimMotB is consistent with well-folded domains connected by a partially flexible, compact linker

**Figure 4:**
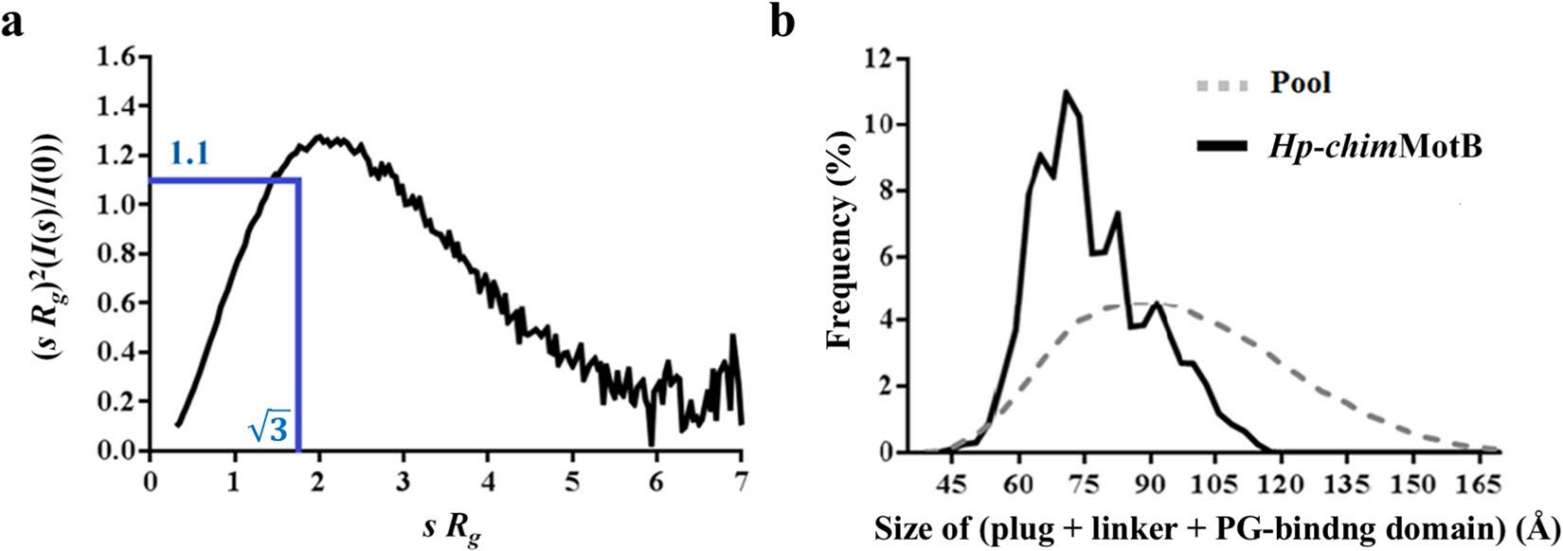
Assessment of *Hp*-*chim*MotB’s flexibility based on the SAXS data. (**a**) Dimensionless Kratky plot, with the intersection of the two blue lines indicating the position of the curve apex (with the value of 1.1) for a theoretical compact globular protein, which occurs at *sR*_*g*_ = √3 (curve not shown). (**b**) *D*_*max*_ distribution for the periplasmic moiety of the EOM-generated *Hp*-*chim*MotB models. The dotted line represents the initial random pool of structures, whereas the solid line represents the ensemble of models selected from this pool which together best match the experimental scattering intensities.

In order to assess the folding state and flexibility of *Hp*-*chim*MotB in solution, the 0.25 mg mL^−1^ SAXS data was analysed on a dimensionless Kratky plot^35,36^ with the axes (*sR*_*g*_)^2^(*I*(*s*)/*I*(0)) versus *sR*_*g*_ (Fig. 4a). On this plot, the theoretical curve of a compact globular protein would be bell-shaped, reaching its apex (1.104) when $s{R}_{g}=\sqrt{3}$. For partially disordered proteins the dimensionless Kratky plot starts trending downward at *sR*_*g*_ values higher than $\sqrt{3}$, but never returns to 0. The higher the *sR*_*g*_ value at the apex of the curve, the greater the flexibility and disorder of the protein^35,36^. For unfolded proteins the curve typically rises to a plateau and then does not change with increasing *sR*_*g*_. The curve calculated for *Hp*-*chim*MotB was broader than the theoretical curve of a typical globular protein, reaching its apex (1.26) at *sR*_*g*_ = 2, and beginning to plateau when *sR*_*g*_ = 5.5 (Fig. 4a). This plot indicated that *Hp*-*chim*MotB contains well-folded domains connected by a partially flexible linker.

The interdomain flexibility of *Hp*-*chim*MotB was assessed using the ensemble optimisation method (EOM)^34,37^. A pool of structures was generated by treating the N-terminal leucine zipper/plug coiled coil region and the C-terminal domain as rigid bodies (modelled based on the known X-ray crystal structures) and connecting them by a linker modelled in 10,000 random yet stereochemically plausible conformations. The distribution of the *D*_*max*_ values was analysed across the ensemble of the pool structures that, when averaged, showed least discrepancy with the experimental data. This analysis revealed that the values for the distance spanned by the plug, linker and C-terminal domain (*i*.*e*. the components that make up the periplasmic portion of MotB), calculated by subtracting the length of the leucine zipper (35 Å) from the *D*_*max*_, fell in the range between 50 and 115 Å (Fig. 4b). Such a broad distribution of distances spanned by the periplasmic moiety indicated that the linker of *Hp*-*chim*MotB exists in a variety of conformations in solution, from compact to elongated. Of note, the most frequently observed conformers in the selected ensemble, contributing ~39% to the total scattering, were compact models in which the periplasmic moiety spanned 62–75 Å, (Fig. 4b). Extended structures were also found in this ensemble. However, extended EOM models were observed at progressively lower frequencies as the linker became more elongated. In fact, only 9% of the selected ensemble models adopted conformations where the periplasmic moiety spanned 100–115 Å (Fig. 4b). This data demonstrates that in *Hp*-*chim*MotB, the compact conformation of the linker is preferable to the extended one.

### Atomic model of MotB generated using shape reconstruction and molecular modelling against the *Hp*-*chim*MotB SAXS data

**Figure 5:**
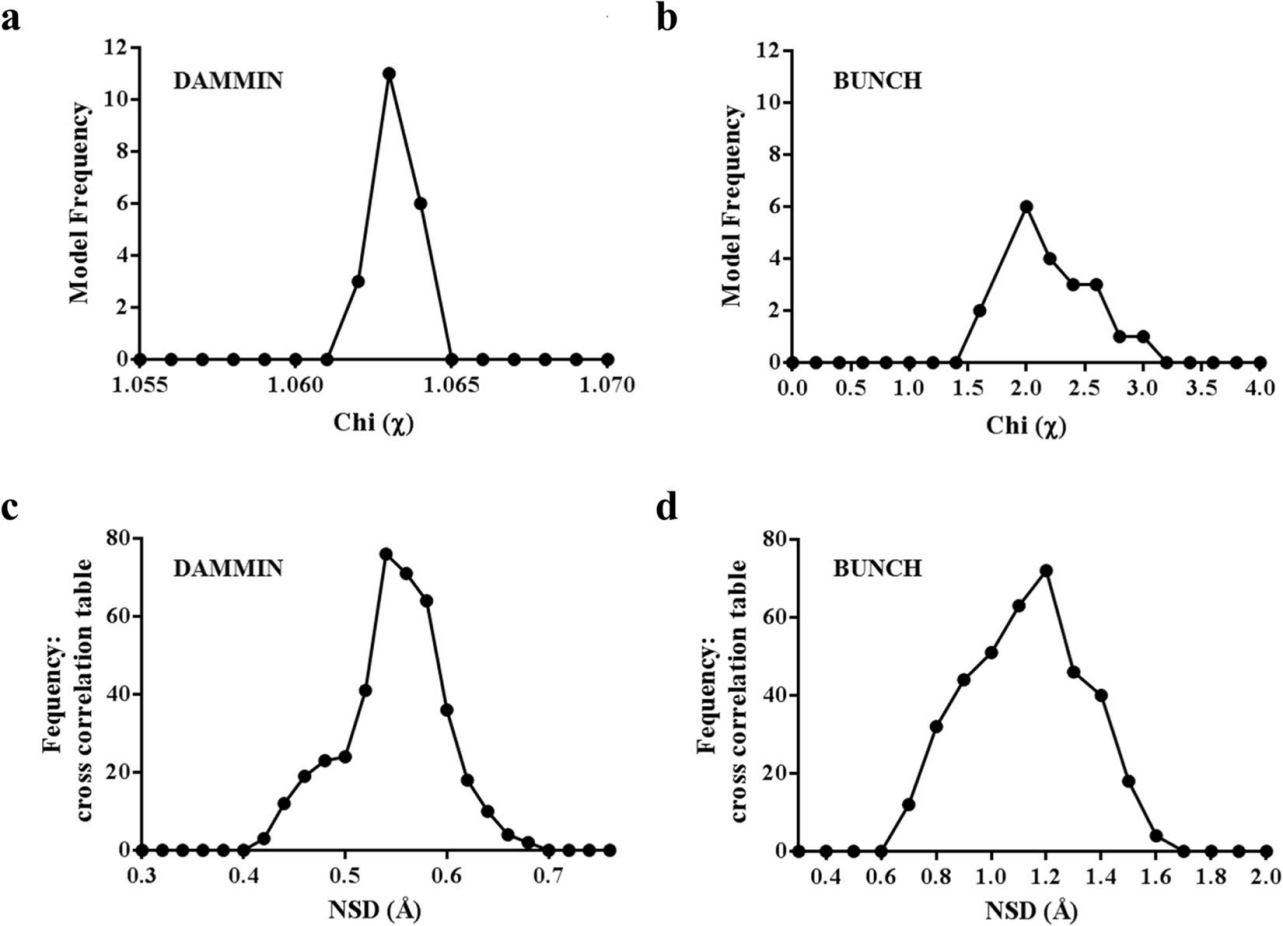
Statistical comparison of the molecular models generated for *Hp*-*chim*MotB and their fit to the experimental SAXS data. χ values calculated for the (**a**) DAMMIN^38^ (*D*_*max*_ = 118 Å) and (**b**) BUNCH^39^ (*D*_*max*_ = 117 Å) models. NSD within the sets of models calculated using (**c**) DAMMIN and (**d**) BUNCH.

**Figure 6:**
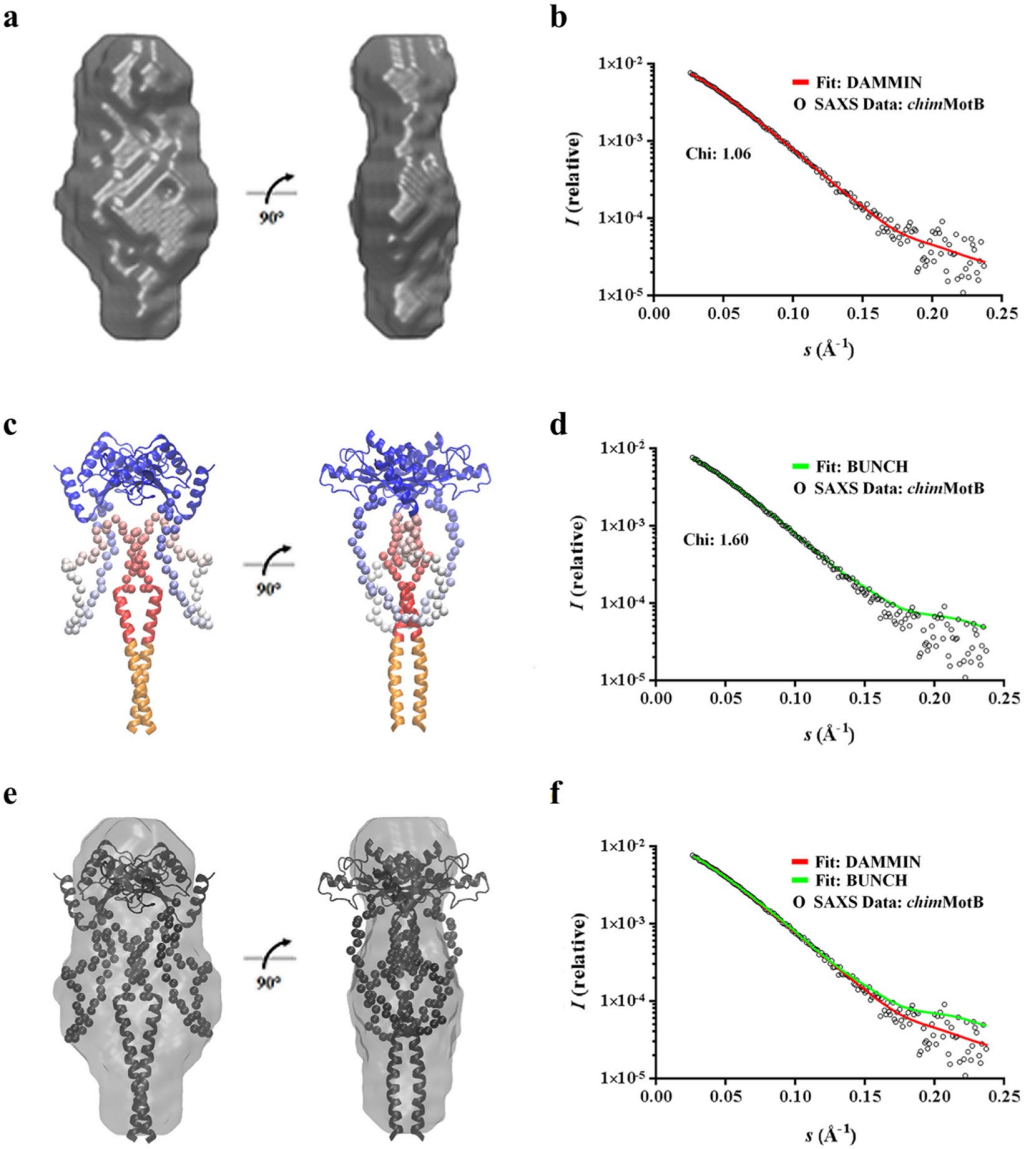
Molecular models of *Hp*-*chim*MotB calculated from the SAXS data. (**a**) Consensus dummy atom model (DAM) envelope of *Hp*-*chim*MotB. (**b**) Comparison of the experimental SAXS data with the theoretical scattering intensities calculated from one of the DAMs within the group used to generate the consensus envelope. (**c**) Selected BUNCH^39^ model of *Hp*-*chim*MotB, with the C-terminal domain (*Hp*-MotB-C, PDB ID: 3CYP^21^) shown in blue and the leucine zipper and plug helices shown in orange and red, respectively (same colour scheme as in Fig. 1a). The dummy atom residues of the linker are coloured in gradient from red (N-terminus) to blue (C-terminus). (**d**) Comparison of the experimental *Hp*-*chim*MotB SAXS data with the theoretical scattering intensities calculated from the selected BUNCH model. (**e**) Superimposition of the consensus dummy atom model (DAM) envelope of *Hp*-*chim*MotB shown in (**a**) (grey) and the selected BUNCH model shown in (**c**) (black) illustrating good agreement between the two approaches (**f**) Comparison of the theoretical scattering intensities calculated from the selected BUNCH model (same as in (**d**), green) with those from one of the DAMs (same as in (**b**), red). The experimental scattering intensities are also shown as open circles.

The DAMMIN algorithm^38^ was employed to generate 20 *ab initio* dummy-atom models (DAMs), all of which had an excellent fit with the experimental data (discrepancy factor χ of between 1.062 and 1.064 (Table 2, Fig. 5a). Figure 6b illustrates agreement between the theoretical scattering intensities of a representative DAM and the experimental data. The mean normalised spatial discrepancy (NSD) between the 20 DAMs, a quantitative ‘dissimilarity’ measure, was 0.55 ± 0.02, which demonstrated good agreement between the models in the set confirming the reliability of the solution (Table 2, Fig. 5c). Only one of the 20 models had an NSD two standard deviations above the mean; that model was discarded before the DAMs were aligned and averaged. The resulting consensus shape is shown in Fig. 6a. The envelope is moderately elongated and has the dimensions of 59 Å × 41 Å × 113 Å. The width of the central lobe (59 Å) is larger than that of the ‘top’ (39 Å) and ‘bottom’ (21 Å) lobes. The size and shape of the ‘top’ lobe are close to those of the crystal structure of *Hp*-MotB-C ^21^ (PDB ID: 3CYP), suggesting that the ‘top’ lobe represents the C-terminal domain of the chimera. Taking into account the known topology of MotB, the two TM helices of which are mimicked by the leucine zipper in *Hp*-*chim*MotB, the smaller (‘bottom’) lobe at the opposite end likely corresponds to the leucine zipper/plug coiled coil, with its N-terminus represented by the protrusion at the bottom end, and its C-terminus (the plug) extending into the central lobe (Fig. 6a,c and e). The remainder of the central lobe likely represents the spectrum of different conformations adopted by the linker.

**Table 2:** Summary of statistics for two modelling regimes used to generate models of *Hp-chim*MotB.

Modelling method	Maximum dimension *D*_*max*_ (Å)	Best χ fit with experimental data	Number of repetitions	Model variation (NSD)
DAMMIN	118	1.06	20	0.55 ± 0.02
BUNCH	117	1.60	20	1.18 ± 0.13

In order to determine the spacing between the plug and the C-terminal domain and explore the range of possible conformations adopted by the linker, BUNCH^39^ was used to generate individual atomic models of *Hp*-*chim*MotB and analyse their fit to the experimental SAXS data. The spacing between the plug and the C-terminal domain was varied to test a range of molecule sizes centred around the P(*r*)-derived *D*_*max*_ value of 118 Å, and for each spacing value, 20 BUNCH models with *P*2 symmetry were constructed. The C-terminal domain was oriented such that the helices at the dimer interface faced outwards (towards PG), consistent with the previous models for *H*. *pylori* MotB in the PG-binding-competent conformation^23^. The probable conformations of the linker were identified by using the simulated annealing procedure and sorted according the discrepancy factor χ. This analysis showed that the set of 20 models with the *D*_*max*_ = 117 Å had the lowest average χ value (data not shown) and contained the conformer (shown in Fig. 6c) that had the lowest individual discrepancy factor (χ = 1.6), and therefore, the best fit to the experimental data, out of all tested structures. Similar calculations performed with the C-terminal domain modelled in the *Hp*-MotB-C/L_90_, rather than *Hp*-MotB-C, conformation yielded higher χ values (>2.1) for all tested models. This result confirms that the C-terminal domain in the chimera adopts a conformation similar to that seen in the *Hp*-MotB-C crystals, which is in line with the FRET measurements and PG-binding assay.

Figure 6d illustrates agreement between the theoretical scattering intensities of the best BUNCH atomic model (shown in Fig. 6c) and the experimental data. Superposition of this model with the consensus DAM envelope using SUPCOMB^40^ shows a close match in shape (Fig. 6e), confirming good agreement between the outcomes of the two different approaches. The atomic model suggests that the mimic of full-length MotB is a relatively compact molecule, where the linker is structured and forms a subdomain that occupies the region between the plug and the C-terminal domain, as well as the area around the plug (Fig. 6c). A significant part of the linker in the model folds against, and forms extensive stabilising interactions with, the parallel coiled coil of the plug.

## Discussion

The stator ring of the bacterial motor is a dynamic structure, with individual units dissociating from the basal body and being substituted with new ones from a pool of ~200 inactive units diffusing in the membrane around the motor^9^. The inactive units in the pool do not bind to the PG layer and have their proton-conducting channels closed, with the hydrophobic faces of the two amphipathic plug helices embedded into the cytoplasmic membrane^28^. Our previous studies suggested that the linker suppresses the PG-binding activity of *H*. *pylori* MotB by folding against the conserved core and stabilising the form of the MotB dimer in which the conserved PG-recognition residues of MotB are buried and the juxtaposition of the two glycan-binding grooves is not optimal for binding to PG^23,25^. Furthermore, we demonstrated that when interactions between the linker and the core domain are lost, the two conserved core domains reorient, aligning the glycan-binding grooves for insertion into the PG mesh, and the PG-binding sites become fully exposed^23^. However, it remained unknown if the linker region of *H*. *pylori* MotB plays a role beyond inhibition of PG-binding activity of stator precomplexes.

Here we have addressed this question by analysing the structure of a soluble variant of full-length *H*. *pylori* MotB locked in its active conformation. Since the opening of the proton channel is known to involve detachment of the two plug helices of the MotB dimer from the cytoplasmic membrane and their association into a parallel coiled coil^28^, we have locked the plug helices in a parallel coiled coil state by fusing them in register with a leucine zipper that replaced the two parallel transmembrane helices of MotB. Fluorescence resonance energy transfer measurements, combined with PG-binding assays and fitting of the crystal structures of MotB fragments to the small angle X-ray scattering (SAXS) data revealed that the protein’s C-terminal domain adopts a PG-binding-competent conformation. This result is consistent with the view that stabilising the dimeric coiled coil form of the plug helix primes the C-terminal domain of *H*. *pylori* MotB for binding to PG, which suggests that, upon assembly of the stator complex into the motor, association of the two plug helices into the dimeric coiled coil may occur concurrently with reorientation of the two C-terminal domains with respect to each other to align their PG-binding sites.

Our analysis of the SAXS data was consistent with the predominant population of molecules having two well-folded domains (transmembrane helix/plug and C-terminal domain) connected by a partially flexible, compact linker. Shape reconstruction and molecular modelling against the SAXS data showed that the linker forms a subdomain occupying the space between the plug and the C-terminal domain, as well as the area around the plug. Modelling suggests that the linker subdomain effectively ‘clamps’ the coiled coil of the plug, thus stabilising the activated form of the protein. *In vivo*, these interactions between the linker and the plug in its coiled coil form likely stabilise the open state of the proton pore within the MotA/MotB stator complex, playing an important role in (*i*) activation of the MotA/MotB stator complex, (*ii*) maintaining it in the active state, and/or (*iii*) force generation.

Thus, our structural analysis suggests that the MotB linker plays a crucial role both in the inhibition of PG-binding activity of inactive stator precomplexes and in the stabilisation of the activated form of MotB. This study adds to the mounting evidence that the linker region is mechanistically important for MotB function^22,23,41^. For example, it has been reported previously that stator assembly defects were observed in *Salmonella* MotB mutants where part of the linker (∆(72–100), ∆(121–130), ∆(131–140)) was deleted^42,43^, suggesting that the linker assists in the assembly of the stator around the rotor.

Although it is not currently known what energises dissociation of the plug helices from the cytoplasmic membrane and their re-arrangement into the parallel coiled coil, it has been suggested that in *Salmonella* and *E*. *coli*, FliL assists the release of the plug helices from the membrane and thus helps the stators to dock with the rotor^16^. Furthermore, it has been proposed that in *R*. *sphaeroides*, motor components FliL and MotF promote the opening of the proton channel through FlgT, which interacts with MotB and triggers the release of the plug^17–19^, although a similar role for the existing homologues in *H*. *pylori* has not yet been identified.

In addition, our analysis demonstrates that although the MotB linker adopts mostly compact conformations, extended conformations (corresponding to the full molecular length of MotB of up to ~150 Å) are also present, albeit in smaller amounts. This is the first direct experimental evidence of the plasticity of the linker connecting the plug and the C-terminal domain of MotB. It is significant because the linker’s ability to extend/contract is thought to form the mechanistic basis for mechanosensitivity of stator units^11^ and to assist the assembly of the stator around the rotor where the distance between the membrane and the PG layer is not uniform.

Although we did not model *Hp*-*chim*MotB in its extended conformations, it is apparent from the geometry of the compact model that, upon extension, the linker would lose its interactions with the plug and could, in principle, fold against the core domain. However, we have previously shown that folding of the linker against the core stabilizes a distinctly different dimeric configuration of the C-terminal domain (represented by the structure of *Hp*-MotB-C/L_90_), that is not competent for binding to PG. We did not see any evidence of that alternative dimer conformation present in *Hp*-*chim*MotB. Indeed, the single symmetric Gaussian shape of the distance distribution obtained from the TR-FRET data (Supplementary Fig. S1) suggested that, both in compact and elongated forms of the protein, the C-terminal domain adopts the same conformation that is able to bind PG. Thus, in all of its states, the linker connected to the open plug appears to be conformationally restrained from associating with the core domain, which ensures that once the plug is open, the activated MotB can extend and contract without losing its ability to bind PG.

## Methods

### PG isolation and purification

*H*. *pylori* strain 26695 was cultured for 48 hrs at 37 °C on GC agar (Oxoid, Basingstoke, UK) supplemented with proteose peptone, 10% (*v/v*) horse serum (Invitrogen), 5 μg mL^−1^ trimethoprim, 10 μg mL^−1^ vancomycin, 10 μg mL^−1^ nystatin and a mixture of vitamins (Supplementary Information), under microaerobic conditions generated using the CampyGen system (Oxoid). Bacteria were harvested from 80 plates by washing with phosphate buffer saline (PBS), yielding 4.3 g of cell paste. Cells were freeze-thawed and resuspended in 25 mL of ice-cold 10 mM Tris-HCl pH 6.8. The mixture was added dropwise into 28 mL of boiling 8% (*w/v*) SDS in 10 mM Tris-HCl pH 6.8 and boiled for 45 min with stirring. Insoluble PG was pelleted by centrifugation at 200,000 g for 60 min at 25 °C. The pellet was resuspended in 140 mL of 2 M NaCl and incubated for 12 hrs at room temperature in order to remove the material bound to PG *via* ionic interactions. PG was pelleted by centrifugation, washed three times in 140 mL of double-distilled water (ddH_2_O) to remove SDS, and resuspended in 20 mL of buffer containing 100 mM Tris-HCl pH 7.5 and 0.1 mM MgCl_2_. The sample was briefly sonicated to increase the accessibility of the PG sacculi during enzyme treatment, 50 µg µL^−1^ DNase I (Roche) and 50 µg µL^−1^ RNase A (Roche) were added to digest nucleic acids, and the mixture was incubated at 37 °C for 90 min. 1 mg of trypsin was then added to digest proteins embedded in PG, and the mixture was incubated at 37 °C for a further 60 min. The volume of the sample was brought to 140 mL with ddH_2_O, 8% (*w/v*) SDS was added, and the mixture was boiled for 15 min with stirring to inactivate the enzymes. PG was pelleted, washed four times with ddH_2_O and lyophilised. This procedure yielded 10 mg of purified *H*. *pylori* PG. Prior to the binding assay, PG was thoroughly resuspended (with sonication) in 1 mL of 50 mM Tris-HCl pH 7.5 to the concentration of 10 mg mL^−1^.

### Protein purification

*Hp*-*chim*MotB, *Hp*-MotB-C (residues 125-256) and *Hp*-MotB-C/L_90_ (residues 90–256) were expressed and purified according to the previously published protocols^21,23,31^. The K146C mutation was introduced into the *Hp*-*chim*MotB expression vector by Genscript (USA). The *Hp*-*chim*MotB_K146C_ variant was expressed and purified by using the protocol previously employed for *Hp*-*chim*MotB^31^.

### PG-binding assay

*Hp*-*chim*MotB, *Hp*-MotB-C and *Hp*-MotB-C/L_90_ were dialysed overnight against 50 mM Tris-HCl pH 7.5 at 4 °C and centrifuged at 400,000 g for 30 min at 4 °C to remove aggregates. Insoluble PG (1 mg mL^−1^) was incubated, whilst rolling at room temperature for 1 hr, with different concentrations of *Hp*-*chim*MotB (35, 55, 85 µM), *Hp*-MotB-C/L_90_ (50, 80, 120 µM) or *Hp*-MotB-C (30, 80, 120 µM)) in 50 mM Tris-HCl pH 7.5 in a total volume of 50 µL. PG and protein associated with it were then pelleted by centrifugation at 400,000 g for 30 min at 4 °C. Following a wash in 200 µL of the same buffer, the pellet was incubated with 50 µL of 2% (*w/v*) SDS in 50 mM Tris-HCl pH 7.5 for 1 hr at room temperature with rolling in order to release the bound protein. The PG was then pelleted by centrifugation, and the supernatant was transferred into a new tube. In order to determine the amount of protein bound to PG, 15 µL-aliquotes of the supernatant from each assay condition were mixed with 5 µL of 5 × SDS-PAGE sample dye and run on a 15% SDS-PAGE gel. The protein bands on the gel were visualised by Coomassie Brilliant Blue R-250 staining and quantified using ImageJ^44^. Each assay included a control sample without PG, and the amount of protein bound to PG was calculated by subtracting the amount recovered from the insoluble fraction in the absence of PG from the amount of insoluble protein retrieved in the presence of PG.

### Protein labelling and time-resolved fluorescence resonance energy transfer (TR-FRET) measurements

*Hp*-*chim*MotB_K146C_ was buffer-exchanged into the labelling buffer that contained 100 mM sodium phosphate buffer pH 7.4 and 200 mM NaCl. To achieve labelling with both donor and acceptor, first *Hp*-*chim*MotB_K146C_ (8.8 μM) was incubated with 5.3 μM donor (([(5-({2-[(iodoacetyl)amino]ethyl}amino)naphthalene-1-sulfonic acid) (IAEDANS)-C2-maleimide, Invitrogen) at 4 °C for 12 hrs, then protein was incubated with 18 μM acceptor ((4-((4-(dimethylamino)phenyl)azo)benzoic acid) (DABCYL)-C2-maleimide, Anaspec) at 4 °C for 2 hrs. The unreacted label was removed using a Zeba size-exclusion spin column (Pierce), and the protein was buffer-exchanged into 100 mM sodium acetate pH 4.6 and 200 mM NaCl. The concentration of protein before and after labelling was determined using Bradford assay (Bio-Rad Laboratories, Hercules, CA). The efficiency of double labelling was determined from the fits of the experimental data using parameter X_DA_ (Supplementary Eq. S4). Double labelling efficiency varied from 15% to 36% in different preparations.

Time-resolved FRET was measured with the home built transient fluorimeter^45^ equipped with an Applied Photophysics SX-18 stopped flow unit (Leatherhead, UK), passively Q-switched microchip YAG laser (SNV-20F-100, 355 nm, 20 kHz, Teem Photonics, Meylan, France), photomultiplier (H6779-20, Hamamatsu, Middlesex, NJ), and fast digitiser (Acqiris DC252, Agilent, Santa Clara, CA). A 420 nm cut-off filter and a polariser set at the magic angle were used in the detection arm. All experiments were done at T = 20 °C. FRET pair labelled protein solution (8 μM) was loaded in the observation cuvette and the donor fluorescence waveform was acquired by averaging fluorescence transients from one thousand laser pulses. The data from different sample preparations (N = 3) were analysed simultaneously. The analysis of the donor lifetime in terms of interprobe distance is described in the Supplementary Information.

### Small angle X-ray scattering (SAXS) data collection and analysis

*Hp*-*chim*MotB was thoroughly dialysed against buffer containing 100 mM sodium acetate pH 4.6 and 200 mM NaCl, with the dialysis buffer retained in order to determine its contribution to scattering. SAXS measurements were acquired at room temperature on the SAXS/WAXS beamline at the Australian Synchrotron, using a 1 M Pilatus detector (DECTRIS). *Hp*-*chim*MotB (0.25 mg mL^−1^, 0.11 mg mL^−1^ and 0.05 mg mL^−1^) and the respective matching dialysis buffer were exposed to X-rays (λ = 1.03 Å) for 1 sec, as the sample flowed through a 1.5 mm quartz capillary, and scattering data were collected over an *s* range of 0.015–0.5 (camera length = 1.6 meters, *s* is the magnitude of the scattering vector). The resulting 2-D scattering images were radially averaged and normalised to give absolute scattering intensities. After scaling, scattering intensities of the respective buffer and empty capillary were subtracted from the intensities of each *Hp*-*chim*MotB sample using PRIMUS^46^. Data analysis was performed using the ATSAS suite of programs^47,48^. Guinier plots generated with PRIMUS were used to calculate values for forward scattering *I*(0) and radius of gyration (*R*_g_) using low resolution data (*sR*_g_ < 1.3). An indirect Fourier transform of the scattering curve *I*(*s*) calculated by GNOM yielded intraparticle distance distribution function in real space P(*r*).

The *R*_g_ values were consistent throughout each different protein concentration tested. The molecular mass (MW) of *chim*MotB was calculated using the zero scattering angle value *I*(0) on the absolute scale and the known scattering of water as previously described^49^; the partial specific volume of the protein was assumed to be 0.74 cm^3^g^−1^. An estimate of the MW (Da) of the protein samples, calculated by dividing the Porod volume V_p_ (Å^3^) (derived using DATPOROD) by 1.7^34^ gave values close to those calculated from *I*(0) (Table 1). The calculated values for the MW of *Hp*-*chim*MotB were consistent across each concentration, indicating that no aggregation was present. The highest protein concentration (0.25 mg mL^−1^) data in the *s* range between 0.026 and 0.238 nm^−1^ was used for shape reconstruction and modelling.

### Shape reconstruction using *ab initio* methods

Particle shapes were restored from the experimental scattering profile of *Hp*-*chim*MotB using an *ab initio* simulated-annealing-based procedure implemented in DAMMIN^38^. The algorithm fits models to the experimental data *I*(*s*) to minimise the discrepancy between the experimental and calculated scattering curves (χ^2^). The *D*_*max*_ and *R*_*g*_ values derived from the P(*r*) analysis were used. The starting model contained 6337 dummy atoms densely packed into a sphere of a diameter equal to *D*_*max*_ (118 Å). Modelling was restrained to prolate shapes with a two-fold axis of symmetry along the direction of anisometry, as *Hp*-*chim*MotB exists as a homodimer in solution^31^. Twenty simulations were performed, which generated very similar, but not identical, shapes. An averaged filtered structure was generated to determine common structural features using DAMAVER^50^, and this structure was used as a fixed core in a final run of DAMMIN to refine the averaged model.

### Molecular modelling against scattering data

In order to assess the size distribution and interdomain flexibility within the ensemble of conformations adopted by *Hp*-*chim*MotB in solution, the SAXS data was analysed using the ensemble optimisation method (EOM)^34,37^. Firstly, RanCh (random chain generator) was used to generate a pool of 10,000 possible *Hp*-*chim*MotB dimer models by treating the N-terminal leucine zipper/plug coiled coil region and the C-terminal domain as rigid bodies and connecting them by a non-clashing linker in random yet stereochemically reasonable conformations. The N-terminal leucine zipper/plug coiled coil region was modelled using the coordinates of a long homodimeric coiled coil observed in the crystal structure of the GCN4 zipper from *S*. *cerevisiae* fused with human vimentin coil 2B fragment^51^ (PDB ID: 1GK6, residues 355–393) as a three-dimensional template. Residues 355–379 of the template, matching in sequence with residues 1–25 in *Hp*-*chim*MotB, were left intact, whilst residues 380–393 were individually substituted to match the sequence of the MotB plug helix 41–54 (residue numbering 26–39 in *Hp*-*chim*MotB) by using the simple mutate function in COOT^52^. The previously published crystal structure of *Hp*-MotB-C^21^ (PDB ID: 3CYP, residues 119–251) was used to model the C-terminal domain. All models in the pool had a two-fold symmetry axis going along the N-terminal coiled coil and C-terminal domain, consistent with the symmetry observed in the corresponding crystal structures. The theoretical scattering intensities of each model were calculated using CRYSOL^53^. GAJOE (Genetic Algorithm Judging Optimisation of Ensembles)^34,37^ was then employed to select an ensemble of the pool structures that, when averaged, had similar theoretical scattering intensities to the experimental data. This algorithm was run 100 times, and the ensemble that showed least discrepancy with the experimental data was selected for the analysis of the *D*_*max*_ distribution.

The BUNCH algorithm^39^ was employed to generate individual models of *Hp*-*chim*MotB with the best fit to the experimental SAXS data. The models with *P*2 symmetry were assembled from the known crystal structures of the N-terminal and C-terminal fragments connected *via* a dummy-reside linker as described above, with the following modifications. Two reported conformations of the dimeric C-terminal domain, represented by the crystal structures of *Hp*-MotB-C/L_90_ (PDB ID: 3S0H) and *Hp*-MotB-C (PDB ID: 3CYP), were tested. The positions of both the N-terminal leucine zipper/plug coiled coil and the C-terminal domain were fixed while the simulated annealing procedure was used to find the probable conformations of the linker that would be in agreement with the experimental data. In separate BUNCH runs, the spacing between the leucine zipper/plug and the C-terminal domain was varied between 42 and 47 Å in 1 Å increments, such that the resulting *D*_*max*_ values of the models (115–120 Å) were centred around the P(*r*)-derived *D*_*max*_ value of 118 Å. For each of the six different spacing values, 20 BUNCH models were calculated and their quality was assessed by calculating the χ value between the scattering intensities of the model and the experimental data.

### Data availability

The 3D coordinates of the model structure of *Hp*-MotB-C generated during the current study are available from the corresponding author on request.

## Electronic supplementary material

Supplementary Information
