# Supplementary material for: Structural analysis of variant of Helicobacter pylori MotB in its activated form, engineered as chimera of MotB and leucine zipper
Source: Sci Rep. 2017 Oct 18;7:13435. doi: 10.1038/s41598-017-13421-0 (PMC5647336; doi:10.1038/s41598-017-13421-0)
Supplement: Supplementary file 1 — Supplementary Information [file 41598_2017_13421_MOESM1_ESM.pdf]

Supplementary Information

Structural analysis of variant of *Helicobacter pylori* MotB in its activated form, engineered as chimera of MotB and leucine zipper.

Daniel A. Andrews, Yuri E. Nesmelov, Matthew C. Wilce and Anna Roujeinikova

Vitamins in *H. pylori* culture. A mixture of vitamins was used to supplement the growth of *H. pylori* strain 26695 on GC agar plates. It was prepared as a 100× stock solution, by combining solutions A and B and making up the volume to 1000 mL with ddH_2O.

Solution A (total volume made up to 500 mL with ddH_2O):

Dextrose (*D*-glucose)	100 g
L-Glutamine	10 g
L-Cysteine	26 g
Cocarboxylase	0.1 g
Ferric nitrate ($Fe(NO_3)_3$	0.02 g
Thiamine-HCl	0.003 g
p-Aminobenzoic acid	0.013 g
Nicotinamide adenine dinucleotide (NAD+)	0.25 g
Vitamin B12 (cobalamin)	0.01 g

Solution B (add 300 mL of ddH2O and 15 mL HCl to dissolve components):

L-Cystine	1.1 g
Adenine	1.0 g
Guanine-HCl	0.03 g
L-arginine	0.15 g
Uracil	0.5 g

TR-FRET data analysis. In the TR-FRET experiment, we observe the waveforms of the fluorescence of donor-acceptor labeled protein and the fluorescence of donor-only labeled protein. The acceptor (Dabcyl) is not fluorescent, and we do not expect to detect fluorescence from the acceptor. Therefore, the waveform of observed transient fluorescence is fitted as a sum of two components, convoluted with the instrument response function (determined from the light scatter). The parameters of donor fluorescence are determined in a separate experiment with donor-only labeled protein, using Eq. S1:

$$F_D(t) = \sum_{i=1}^{2} A_i \exp(-t/\tau_{Di}), \qquad \text{Eq. S1}$$

where τ_{Di} are the donor-only fluorescence lifetimes.

The interprobe distance and distance distribution $\rho(r)$ are determined in the experiment with donor-acceptor labeled protein, using Eq. S2. The donor fluorescence parameters determined in the previous experiment were kept fixed.

$$F_{DA}(t) = \int_0^{+\infty} \rho(r) \cdot \sum_{i=1}^{2} A_i \exp\left\{\left(-\frac{t}{\tau_{D_i}}\right) \cdot \left(1 + \left[\frac{R_0}{R}\right]^6\right)\right\} dr, \qquad \text{Eq. S2}$$

where R_0 = 4 nm is the Förster distance for IAEDANS-Dabcyl pair and $\rho(r)$ is a probability density function to account for the interprobe distance distribution. We used a Gaussian distribution as a probability density function:

$$\rho(r) = \frac{1}{\sigma\sqrt{2\pi}} \exp\left(-\frac{(r-R)^2}{2\sigma^2}\right), \qquad \text{Eq. S3}$$

where σ is the standard deviation, determining the interprobe distance distribution, and R is the position of the distribution maximum.

The mole fraction of the donor-acceptor labeled protein, and the corresponding interprobe distance and distance distribution are therefore determined from the fit of experimental data according to Eq. S4, with all previously determined parameters fixed.

$$F_{total}(t) = X_D \cdot F_D(t) + X_{DA} \cdot F_{DA}(t), \quad X_D + X_{DA} = 1, \qquad \text{Eq. S4}$$

In this equation, X_D and X_{DA} are the mole fractions of donor-donor labeled protein (X_D), and donor-acceptor labeled protein (X_{DA}).

The Förster distance, R_0, was calculated as

$$R_0 = 9786[J(\lambda)\kappa^2\eta^{-4}Q_D]^{1/6}, \qquad \text{Eq. S5}$$

where λ is the wavelength, $J(\lambda)$ is the spectral overlap integral between normalized donor emission spectrum $F_D(\lambda)$ and the acceptor absorption spectrum $\varepsilon_A(\lambda)$, $\kappa^2 = 2/3$ is the probes orientation factor, η =1.4 is the refraction index of the medium, Q_D is the donor quantum yield[1].

Analysis of transient time-resolved fluorescence data was performed using software package FargoFit, designed by I. Negrashov, that executes global least-square fitting of multiple time-resolved fluorescence waveforms using different kinetic models with ability to link fitting parameters between waveforms.

Supplementary References

1. Agafonov, R.V., et al., Structural dynamics of the myosin relay helix by time-resolved EPR and FRET. *Proc. Natl. Acad. Sci. U. S. A.* **106**, 21625-21630 (2009).

Supplementary Figures

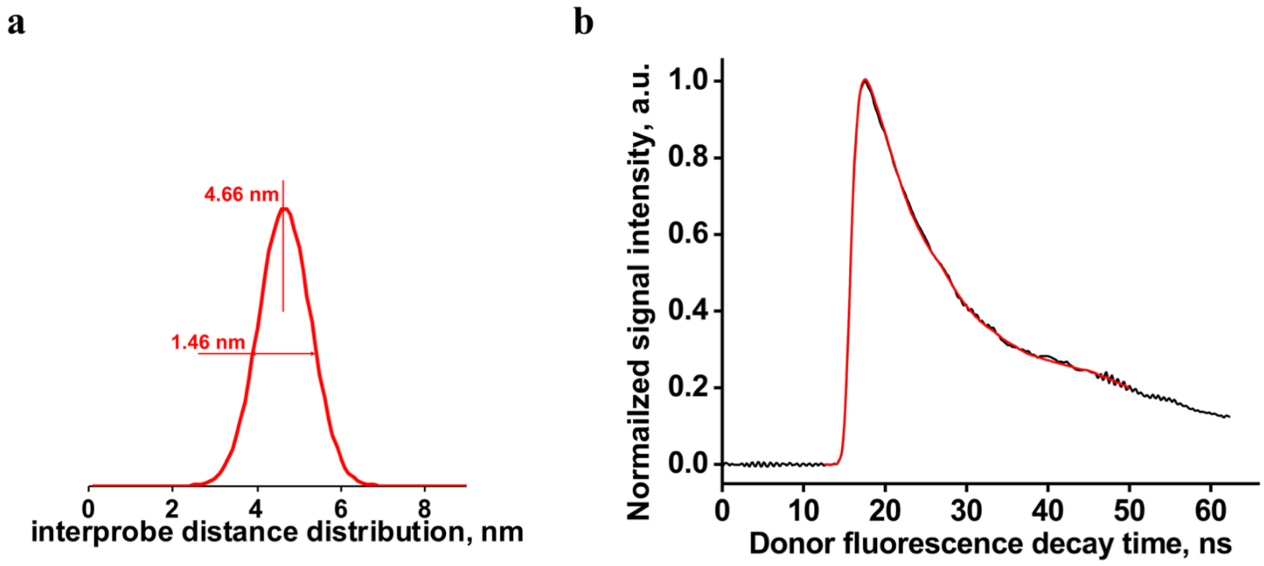

Figure S1. (**a**) Gaussian distance distribution of the FRET pair within the dimer, obtained by fitting fluorescence signals. The peak position is 4.66 nm with the FWHM 1.46 nm, corresponding to the interprobe distance 4.66 ± 0.62 nm. (**b**) Transient fluorescence signal of donor–acceptor-labeled dimer and fit to the Eq. S4.
